# Atomistic Investigation of Anisotropic Nanoindentation Behavior of Nanotwinned Aluminum Containing Inclined Twin Boundaries

**DOI:** 10.3390/nano8090695

**Published:** 2018-09-06

**Authors:** Yuan Liu, Yanfeng Duan, Junjie Zhang

**Affiliations:** 1 School of Astronautics, Harbin Institute of Technology, Harbin 150001, China; liuyuan_hit@hit.edu.cn; 2 Research Department of Structure Design and Simulation, Graduate School of Hubei Aerospace Technology Academe, Wuhan 430040, China; duanyanfeng1986@163.com; 3 Center for Precision Engineering, Harbin Institute of Technology, Harbin 150001, China

**Keywords:** aluminum, nanoindentation, twin boundary, inclination angle, molecular dynamics

## Abstract

Nanotwinned metals exhibit superior mechanical properties due to unique dislocation–twin boundary interactions. In the present work, we elucidate the microscopic deformation mechanisms and their correlations with the macroscopic mechanical response of nanotwinned Al containing inclined twin boundaries under nanoindentation by means of molecular dynamics simulations. The effect of twin boundary orientation with respect to the indented surface on the nanoindentation is evaluated. Simulation results reveal that dislocation slip, dislocation–twin boundary interaction, and twin boundary migration operate in parallel in the plastic deformation of nanotwinned Al. The inclination angle of twin boundaries with respect to indented surface has a strong influence on the interaction between individual deformation modes, which in turn leads to the anisotropic indentation behavior of nanotwinned Al.

## 1. Introduction

Aluminum is one of the most widely used key materials for aerospace applications due to its low density, high strength, high wear resistance, and good workability. In particular, mechanical properties play a critical role in determining the performance of aluminum-based components and devices. The nanoindentation technique has been widely used to derive intrinsic mechanical properties, such as Young’s modulus and hardness, of different types of materials at the micro/nanoscale [1,2].

With the development of hierarchically structured materials, interface engineering has been playing an important role in tailoring mechanical properties of materials. For instance, nanotwinned metals containing imbedded twin boundaries (TBs) show extraordinary properties of high electrical conductivity, high strength, and considerable ductility. While strengthening originates from TB blocking dislocation motion, ductility results from TB acting as a dislocation source or from TB migration itself [3,4,5,6]. For aluminum in particular, Bufford et al. reported a high work-hardening capacity and plasticity in twinned Al [7].

The alignment of TBs plays a central role in determining the mechanical behavior of twinned metals. Wei et al. found that the plasticity of twinned metallic nanowires can be dominated by TB-associated mechanisms by designing the inclination angle of TBs [8]. Stukowski et al. showed that the dislocation–TB intersection in nanotwinned Cu under uniaxial tension can be altered by the TBs’ orientation with respect to the loading direction [9]. Brown et al. showed that the plasticity in twinned Cu nanopillars greatly depends on the TBs’ orientation with respect to the loading axis [10]. Zhang et al. investigated the effect of the inclination angle on the nanoscratching behavior of nanotwinned copper, and found a critical inclination angle of 26.6° for the lowest yield strength and the highest friction coefficient [11]. However, there is rather limited work focusing on the effect of the inclination angle of TBs on the mechanical properties of aluminum.

Therefore, in the present work, we perform molecular dynamics (MD) simulations to investigate the deformation mechanisms of nanotwinned Al containing inclined TBs under nanoindentation. The microscopic deformation mechanisms of materials are elucidated and are correlated with the macroscopic response of nanoindentation. We further investigate the effect of the inclination angle of TBs with respect to the indented surface on the nanoindentation process. 

## 2. Simulation Method

**Figure 1:**
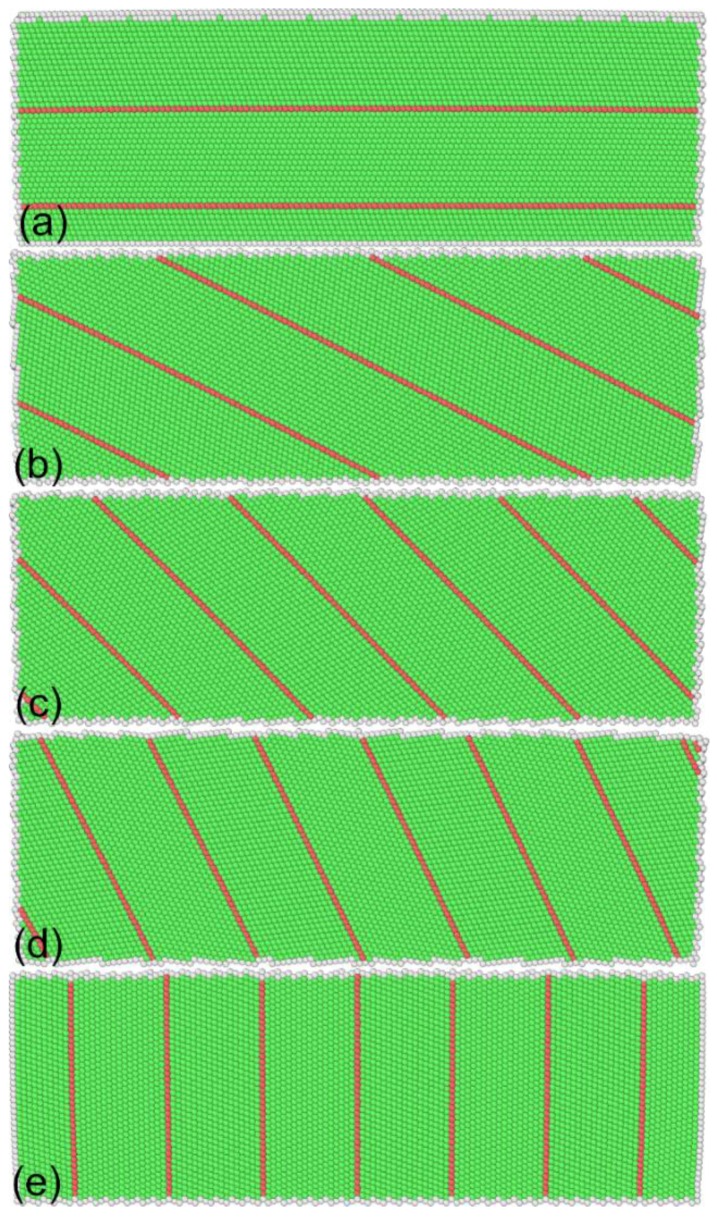
Atomic structures of nanotwinned Al containing differently inclined twin boundaries (TBs). Inclined angle of TBs: (**a**) 0°; (**b**) 26°; (**c**) 45°; (**d**) 64°; and (**e**) 90°. Atoms are colored according to their common neighbor analysis (CNA) values.

Figure 1 shows the atomic structures of nanotwinned Al specimens containing differently inclined TBs. Five inclination angles are considered, as 0°, 26°, 45°, 64°, and 90°, respectively. To construct the TBs with different inclination angles, the twinned specimen containing TBs with an inclination angle of 0° is first constructed by periodically changing the atomic arrangement of a single crystalline specimen along the (111) direction. Then, the refereed specimen is rotated with a 2D rotation matrix along the (110) direction. Each specimen has a dimension of 35 nm in length, 12 nm in height, and 35 nm in width, respectively. The Al–Al atomic interactions within the aluminum specimen are described using an embedded atom method (EAM) [12]. The intrinsic stacking fault energy of Al predicted by the utilized EAM potential is 146 mJ/m^2^, which agrees well with the experimental values of 120–144 mJ/m^2^ [13].

Prior to nanoindentation, the as-constructed specimen is subjected to relaxation through the following procedures: First conjugate-gradient energy minimization at 0 K, and then dynamic relaxation at 3 K and under 0 bar for 50 ps using the canonical (constant number of atoms, constant volume and constant temperature, NVT) ensemble. After reaching the equilibrium configuration, the specimen is subjected to nanoindentation using a spherical indenter. The indenter with a radius of 4 nm is modeled by a strong repulsive potential [14]. Nanoindentation is performed in a displacement-controlled mode, that is, the indenter penetrates the specimen surface with a constant velocity of 20 m/s until it reaches a predetermined indentation depth of 2 nm. The common neighbor analysis (CNA) is utilized to distinguish types of lattice defects [15]. The coloring scheme is as follows: Green stands for face-centered cubic (FCC) atoms, red for hexagonal closed packed (HCP) atoms, and gray for free surface and dislocation cores. All the MD simulations are performed using the Large-scale Atomic/Molecular Massively Parallel Simulator (LAMMPS) code with an integration step of 1 fs [16]. Ovito is utilized to visualize MD data and generate MD snapshots [17].

## 3. Results and Discussion

### 3.1. Deformation Mechanisms

**Figure 2:**
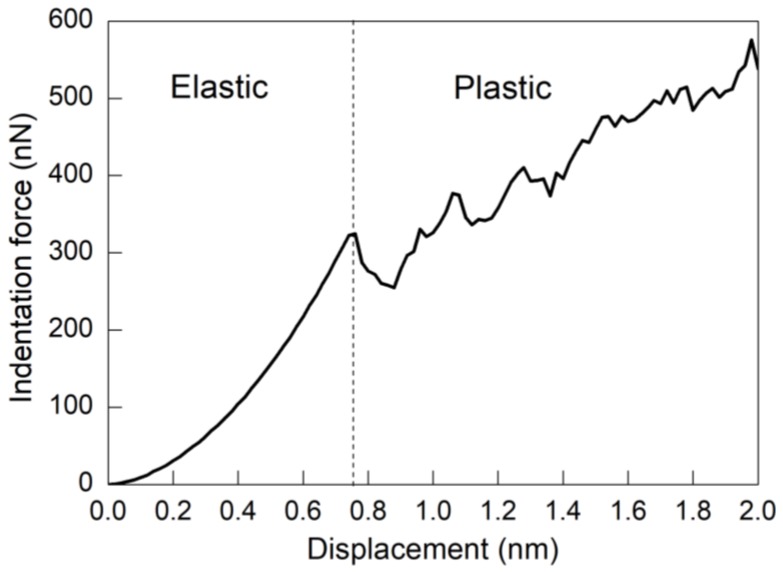
Force–displacement curve of twinned Al with an inclination angle of 0° under nanoindentation.

To obtain the fundamental deformation mechanisms of twinned Al, MD simulation of nanoindentation on the twinned Al with an inclination angle of 0° is first performed. Figure 2 plots the force–displacement curve obtained, which shows that the nanoindentation can be categorized into two stages, as elastic deformation and plastic deformation, respectively. Specifically, Figure 2 shows that the transition from elastic stage to plastic stage is accompanied with a rapid decrease of indentation force, which is corresponding to the pop-in phenomenon observed in the load-controlled nanoindentation tests.

**Figure 3:**
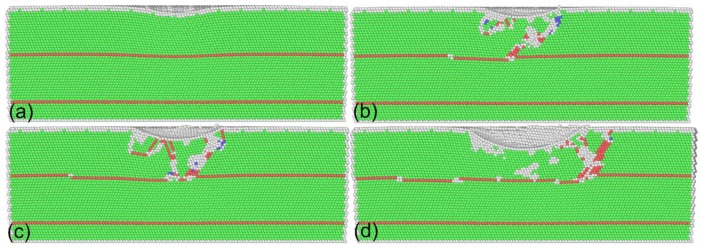
Cross-sectional views of twinned Al with an inclination angle of 0° under different displacements. Displacement: (**a**) 0.72 nm; (**b**) 0.88 nm; (**c**) 1.12 nm; and (**d**) 2.0 nm. Atoms are colored according to their CNA values.

Figure 3 presents MD snapshots of the twinned Al with an inclination angle of 0° at different displacements. It is seen from Figure 2 that there is a rapid increase of indentation force in the elastic stage. Figure 3a shows that, at a displacement of 0.72 nm, the free surface in contact with the indenter is curved, but there is no internal defect generated, indicating that the material is undergoing pure elastic deformation. Upon further advancement of the indenter, a dynamic inspection of deformation behavior shows that the force drop shown in Figure 2 is caused by the emission of dislocations from surface in contact with the indenter. After nucleation, dislocation glides on the (111) slip planes along the (110) slip directions. The avalanche of dislocations releases the strain energy accumulated in the elastic stage, which leads to the force drop. However, the subsequent sliding of dislocations is hindered by the TBs. Figure 3b shows that, at a displacement of 0.88 nm, there is an intersection of lattice dislocations with the horizontal TB. Consequently, the TB is divided into three sections that are connected by twinning partial dislocations, that is, TB migration occurs. 

**Figure 4:**
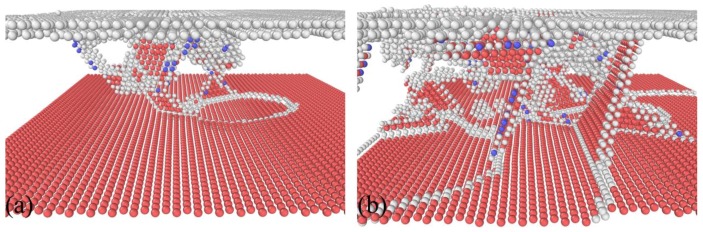
Representative dislocation–TB interactions. Displacement: (**a**) 0.88 nm; (**b**) 1.6 nm. Atoms are colored according to their CNA values, and perfect face-centered cubic (FCC) atoms are not shown.

Figure 4a further presents the instantaneous zoom view of the dislocation–TB interaction at a displacement of 0.88 nm. It is found from Figure 4a that only a portion of TB is migrated to the distance of an atomic layer, and the dislocations are confined within the twin lamellae. The confinement of dislocations by TBs leads to work hardening of the indented material, which is accompanied by the increase of indentation force. Upon further indentation, fresh dislocations are successively nucleated to accommodate the plastic strain induced by the advancement of the indenter. The fluctuation events shown in the force–displacement curve are associated with the successive nucleation events of dislocations. However, dislocation motions are still confined to the first twin lamellae. Furthermore, there are more pronounced interactions between dislocation and TB, as shown in Figure 3c. Figure 4b presents the instantaneous zoom view of the dislocation–TB interaction at a displacement of 1.6 nm. While considerable dislocations in the twin lamellae exist, dislocations are mainly inclined to the TB that is parallel to the free surface. Consequently, multiple TB migrations occur. Figure 3d shows that after the completion of nanoindentation, dislocation density is significantly decreased due to the absorption of dislocations by TBs, and dislocations are completely confined within the twin lamellae. Figure 3d also demonstrates that the migrated TB segments are not recovered. The above results indicate that the plastic deformation of twinned Al under nanoindentation is governed by dislocation slip, dislocation–TB interaction, and TB migration in parallel. A previous study indicated that the mechanical response and dislocation motion in bicrystal Al containing TBs under nanoimprinting are not strongly influenced by the presence of TBs [18].

### 3.2. Influence of Inclination Angles

**Figure 5:**
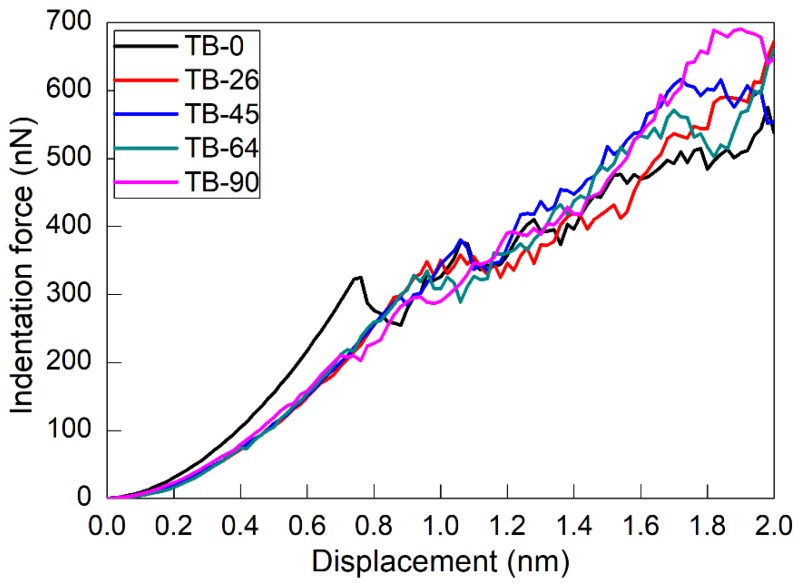
Force–displacement curves of twinned Al containing TBs with different inclination angles under nanoindentation.

MD simulations of nanoindentation on twinned Al with differently inclined TBs are also performed to investigate the influence of the inclination angle on the nanoindentation response. Figure 5 plots the force–displacement curves during nanoindentation for different inclination angles. It is seen that for each inclination angle, the variation of indentation force has similar characteristics: It first increases rapidly in the elastic deformation, then drops at the elastic–plastic transition, and then finally increases with fluctuations in the plastic deformation. We note that the force drop phenomenon is not equally pronounced for different inclination angles, due to intrinsically different crystallographic orientations. However, the force–displacement curves for the inclination angle of 0° show a higher slope than those for the other four angles, indicating a higher Young’s modulus of the twinned Al with an inclination angle of 0°. For the other four inclination angles, although the force–displacement curves in the elastic deformation have approximately the same slopes, the critical displacement and associated critical force for the elastic–plastic transition are different for different inclination angles, as demonstrated in Table 1. Specifically, the inclination angle of 45° has the highest critical displacement and critical force, followed by 26°, 64°, 0°, and 90°. After yielding, the difference in the force–displacement curves for different inclination angles is reduced due to complex deformation behavior.

**Figure 6:**
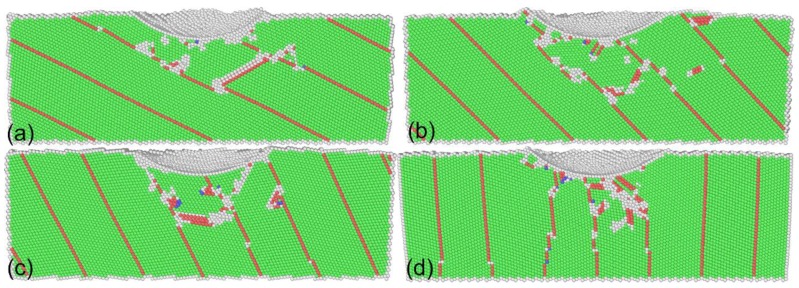
Cross-sectional views of twinned Al with different inclination angles after nanoindentation. Inclination angle: (**a**) 26°; (**b**) 45°; (**c**) 64°; and (**d**) 90°. Atoms are colored according to their CNA values.

**Figure 7:**
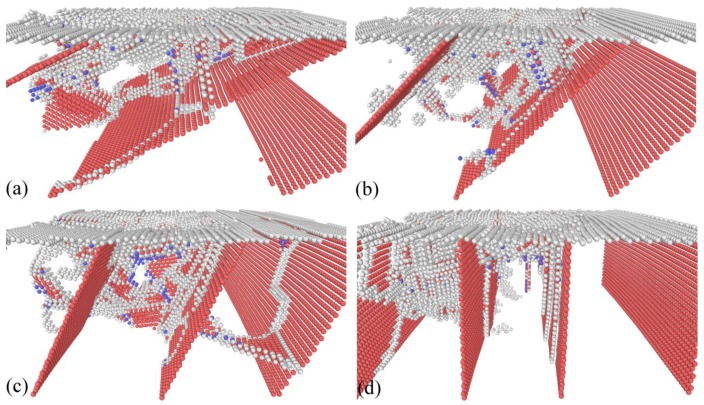
Representative dislocation–TB interactions. Inclination angle: (**a**) 26°; (**b**) 45°; (**c**) 64°; and (**d**) 90°. Atoms are colored according to their CNA values, and perfect FCC atoms are not shown.

Figure 6 presents the cross-sectional views of twinned Al with different inclination angles after nanoindentation. Figure 7 further presents defect structures of twinned Al with different inclination angles after nanoindentation. As with the inclination angle of 0°, TBs act as barriers for dislocation motions, which also leads to TB migrations. However, the confinement of dislocation within twin lamellas is different for different inclination angles. For the inclination angle of 90°, there are lattice dislocations gliding parallel to TBs, in addition to inclined dislocations. However, only inclined dislocations are observed for the other inclination angles. Consequently, TB migration is more pronounced for the inclination angle of 90° than for the other four inclination angles. For the inclination angles except for 0° in particular, there are lattice dislocations emitting from the migrated position of TBs, indicating that TB acts as a dislocation source, which means dislocation activity is easier to be triggered. This also indicates the lower strength of twinned Al with an inclination angle different from 90°. The above results also indicate that the mean work hardening rate in the plastic regime increases with increasing inclination angle of TBs.

**Figure 8:**
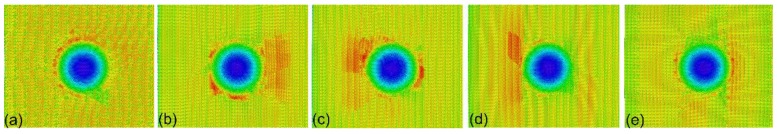
Surface morphology of twinned Al with different inclination angles. Inclination angle: (**a**)0°; (**b**) 26°; (**c**) 45^o^; (**d**)64°; and (**e**) 90°. Atoms are colored according to their atomic heights.

Figure 8 presents the surface morphology of the indented twinned Al after the completion of nanoindentation. It is seen that the surface pile-up around the residual impression is significantly different for different inclination angles. The surface pile-up is most pronounced for the inclination angle of 45°, followed by 26°, 64°, 0°, and 90°. It is indicated that the surface pile-up behavior is closely associated with the strength of the twinned material, which is determined by the alignment of TBs. The propagation direction of surface pile-up is also different for different twinned materials. While there are intersections of TBs with free surface for the inclination angle of 26°, 45°, 64°, and 90°, the propagation of displaced materials is mainly along the intersection direction. However, surface pile-up for the inclination angle of 0° is different.

## 4. Conclusions

In summary, we performed MD simulations to investigate the fundamental deformation mechanisms of nanotwinned Al containing inclined TBs under spherical nanoindentation. It was found that the plastic deformation of nanotwinned Al is dominated by individual deformation modes of dislocation slip, dislocation–TB interaction, and TB migration, the interaction between which is strongly dependent on the inclination angle of TBs with respect to the indented surface. The mean work hardening rate in the plastic regime increases with increasing inclination angle of TBs. Specifically, for an inclination angle of 45°, the critical force and displacement for the elastic–plastic transition are the highest, and the surface pile-up morphology is the most pronounced.

## Figures and Tables

**Table 1:** Critical force and displacement for different inclination angles.

Inclination Angle	Critical Force (nN)	Critical Displacement (nm)
0°	326.5	0.76
26°	348.3	0.96
45°	380.8	1.06
64°	327.1	0.91
90°	209.0	0.70
